# Relapse in Typhoid Fever

**Published:** 1898-03-05

**Authors:** 


### RELAPSE IN TYPHOID FEVER.

In a paper in the *Practitioner* for this month Dr. Bertram Hunt states that of 71 cases of typhoid fever recently treated in University College Hospital, no less than 28—*i.e.*, 40 per cent.—underwent a relapse. This proportion is greatly in excess of the usual frequency, which Murchison gives as 3 per cent. No cause could be discovered for this frequency of relapse, although, as negativing certain assumed causes, the cases were of much interest. For example, much has been said about relapse following cold, exposure, fatigue, or errors in diet; but nearly all these cases occurred while the patients were still in bed, and while they were taking strict typhoid diet; while no relapse was seen in several cases which were tentatively treated on the lines advocated by Barrs, by giving solid food immediately the temperature fell. This shows the small importance of these factors in the production of a true relapse, although they might undoubtedly produce a false relapse or after-fever, which has often been confused with the true condition. Again, these cases were not treated by cold baths, a method which has often been observed to be followed by a greater frequency of relapse.

With but one or two exceptions also the relapses were not seen in the mildest cases, nor was relapse associated with constipation, as has been asserted to be the case, the relapses among the diarrhœa cases being three times as numerous as among those which were the constipated cases, the total number of patients in each group being the same. Dr. Hunt finally points out that, whatever the cause of the reinfection, it would be obviously inoperative unless the immunity produced by the first attack was either incomplete or was produced more slowly in these cases.

Unfortunately, he says, we cannot estimate clinically the grade of the immunity that exists. In many cases which ultimately relapsed the blood serum gave a well-marked agglutinative reaction early in the attack; but this cannot be taken as showing a high degree of immunity, since Widal and others hold that it is not a reaction of immunity. The cause of relapse at present remains a mystery.

